# Endoscopic esophageal stenting for advanced esophageal cancer in Lubaga Hospital, Kampala, Uganda

**DOI:** 10.1186/s13104-022-06236-4

**Published:** 2022-11-01

**Authors:** Michael Okello, Dave Darshit, Esther Patience Nabwire, Anna Ainembabazi Tinka, Sabrina Bakeera-Kitaka, Ponsiano Ocama

**Affiliations:** 1 Department of Anatomy, Makerere University College of Health Sciences., P.O Box 7072, Kampala, Uganda; 2 Department of Surgery, Lubaga Hospital, Kampala, Uganda; 3 Department of Paediatrics, Makerere University College of Health Sciences., Kampala, Uganda; 4 Department of Medicine, Makerere University College of Health Sciences., Kampala, Uganda

**Keywords:** Esophageal cancer, Endoscopy, Stenting, Uganda

## Abstract

**Objective:**

Esophageal cancer is a common malignancy globally. Most patients in sub-Saharan Africa present at advanced stage not amenable to curative therapy. Stenting provides palliation for these patients. In Uganda, many endoscopy units can perform diagnostic endoscopy but only a handful routinely perform endoscopic interventions like stenting. We describe esophageal cancer patients who underwent esophageal stenting intending to highlight its importance in a resource-limited setting.

Endoscopy reports were reviewed for patients who underwent evaluation for esophageal cancer at Lubaga Hospital from December 2014 to March 2022.

**Results:**

315 records of patients with esophageal cancer were reviewed. Male to female ratio was 2:1. 188(60%) patients were 60 years and above. 268 (85%) esophageal lesions were described as fungating, friable or polypoid. 249 (79%) tumors were in mid or distal esophagus. 66% esophageal lesions caused severe luminal obstruction not traversable by the scope. 164 (52%) patients did not opt for stenting due to personal and other reasons. Stenting wasn’t successful in 7 out of the 148 patients who underwent either primary or tandem stenting. Despite 207 (66%) of patients with advanced esophageal cancer presenting with endoscopically non-traversable tumors, endoscopic stenting was still possible with a technical success rate of 95.3%.

## Introduction

Cancer of the esophagus is one of the most aggressive malignancies of the upper digestive tract. In Uganda, cancer of the esophagus is the third commonest malignancy in men and the fourth commonest in women, with an age-standardized incidence rate of 13–14 per 100,000 of the population. It remains a challenging tumor to treat with many patients presenting with advanced disease [1]. Histologically, cancer of the esophagus is composed of two main histologic types- squamous cell carcinoma and adenocarcinoma- with a rising incidence globally. In most resource poor settings, squamous cell carcinoma is still the most common type of cancer of the esophagus [2].

Esophageal obstruction and tracheoesophageal fistula formation are frequent complications. Since the majority of patients present late with unresectable disease, one of the palliative care modalities is the insertion of esophageal stent to relieve the dysphagia, nutritionally optimize the patient prior to chemotherapy and it also helps to minimize the chances of micro-aspirations in patients with grade IV dysphagia. Other options include: dilation, chemical or ablative debulking, and enteral feeding. Endoscopic esophageal stenting involves the insertion of a stent to restore luminal patency. This can be done with or without concomitant fluoroscopic guidance [3]. Stenting is also used for conservative management of esophageal leaks, perforations, stenosis and fistulas. Other modalities used to open the esophageal lumen includes dilatation which involves the passage of an expandable balloon or wire-guided polyvinyl bougies through a malignant stricture and results in immediate expansion of the luminal diameter, often enabling improvement in swallowing [4].

During these interventions, endoscopy is vital. Diagnostic upper gastrointestinal (GI) endoscopy involves the examination of lining of the oropharynx, hypopharynx, esophagus, stomach and the proximal parts of the duodenum usually with a flexible endoscope while interventional endoscopy may involve treating the condition discovered during the diagnostic endoscopy. Interventional endoscopies include esophageal foreign body removal, variceal band ligation, esophageal stenting, polypectomies, endoscopic mucosal resection (EMR), endoscopic submucosal dissection (ESD), peroral endoscopic myotomy (POEM), endoscopic retrograde cholangiopancreatography with or without sphincterotomy or a sphincteroplasty etc. [5, 6]. Endoscopy allows the physician to visualize and biopsy the mucosa [7, 8]. However, these procedures are not commonly performed in many resource limited settings where treatment is composed of pain management. Over the years, we have performed several procedures at Lubaga Hospital.

We reviewed the records of the patients who underwent esophageal stenting for esophageal cancer in Lubaga Hospital, Kampala, Uganda. We hope that the results will inform clinicians in resource-limited settings of the potential benefit interventional endoscopy has in palliation of dysphagia in patients with unresectable esophageal cancer.

## Methods

### Study design

This was a review of endoscopy reports for patients who underwent evaluation for Cancer of the esophagus at Lubaga Hospital from December 2014 to March 2022. Staging thoracoabdominal CT scan for resectability and evaluation by the oncologist and a cardiothoracic surgeon was the pre-requisite for esophageal stenting.

### Study site and setting

The study was conducted at Lubaga Hospital, a private not-for-profit hospital located in the Lubaga division of Kampala capital city of Uganda. This is the second oldest hospital in Uganda founded by catholic missionaries in 1899. The hospital has an endoscopy unit, under the Surgery department that runs both inpatient and outpatient services, as well as diagnostic and interventional endoscopy services. The hospital acquired an endoscopy unit in the year 2014 and also obtained a C-arm for procedures that require fluoroscopic guidance. The hospital performs both diagnostic and some therapeutic procedures including esophageal stenting, variceal banding, ERCP [9], foreign body removal and a number of other procedures both for children and adults. In addition to upper gastrointestinal endoscopy, the unit also performs lower GI diagnostic and therapeutic procedures mostly polypectomies and colonic stenting. The unit receives requests from within the hospital and other hospitals within Kampala and sometimes beyond Kampala city, and an average of 60 endoscopy procedures are performed in a month.

### Endoscopic esophageal stenting procedure

All procedures were done under propofol sedation for stable patients under 60 years of age and are classified by American Society of Anaesthesiologists (ASA) as ASA 1 or 2. Local anaesthesia (xylocaine spray) was used for unstable and elderly patients above 60 years old and those patients irrespective of age that were classified as ASA 3, 4 or above. Barium swallow was not a pre-procedure prerequisite. All patients were stented under endoscopic guidance in absence of fluoroscopy. Upper gastrointestinal (GI) endoscopy was performed until the duodenum and a spring tipped steel guide wire left in situ under direct vision by withdrawing the scope while pushing in the guide wire at the same rate similar to the “Seldinger technique” done during central venous catheter placement. The stent with its delivery system were passed into the esophagus guided by the steel wire. The scope was then re-inserted and the stent deployed across the tumor under direct vision.

For endoscopically traversable cancer, the stent size was determined by length of malignant stricture estimated on markings of the gastroscope. 2 cm allowance was given proximal and distal to the tumor before deployment to minimize occlusion of the proximal or distal ends of the stent by tumor overgrowth.

For endoscopically non-traversable esophageal strictures, spring tipped steel guide wire was passed through the residual tiny orifice under vision followed by esophageal bougienage, to dilate the stricture upto about 1.5–2 cm diameter when the scope can now traverse the stricture. The malignant stricture length could then be determined using the markings on the gastroscope and an appropriate length stent deployed as described above. The procedures were mostly done on outpatient basis. The patients were observed in the recovery room for about 45 min during which they were asked to take a cup (500mls) of warm tea or soup under observation as a rough measure of oral intake. The patient was then allowed home or back to the referring facility if no vomiting or any other complaints. For all these patients, we were inserting non-vascular Ni–Ti alloy self-expanding stents from Changzhou Health Microport Medical Device Co. Ltd Jiangsu, P.R. China. http://www.cz-hmm.com.

### Data collection and analysis

A data abstraction tool collected information on demographics (age, gender), endoscopic findings (tumor location, tumor description, extent of occlusion and interventions).

Data was summarized using descriptive measures. Demographic characteristics were summarized by standard descriptive summaries (percentages for categorical variables such as gender).

## Results

### Participant characteristics

A total of 315 records of patients with an endoscopic diagnosis of esophageal cancer were reviewed. Male to female ratio was 2:1 and 60% of the patients were 60 years and above.

### The description of tumor at endoscopy

Most of the lesions were fungating, friable and polypoid, contributing 85.07% of all the tumors. Gastro-esophageal junction (GEJ) cancer accounted for 7% while Cicatrizing tumors and esophageal nodules accounted for 2.5% and 5.4% respectively. Other descriptions included stenosis and ulceration.

The middle one-third of the esophagus was the most common site of tumor location accounting for 40.3% of the tumors described followed by the distal and proximal esophagus at 38.7% and 14% respectively.

At the time of initial presentation, the level of esophageal obstruction was determined by the ability to traverse through with aid of a scope. 65.71% of the tumors were non-traversable by the scope (Table 1).

**Table 1:** Demographic and esophageal tumor characteristics

Variable	Frequency (N)	Proportion (%)
Gender		
Female	104	33.01
Male	211	66.98
Age group		
20–39	10	3.17
40–59	117	37.14
60–79	147	46.66
> 80	41	13.01
Tumor description		
Cicatrizing tumor	8	2.53
Fungating + Friable + polypoid	268	85.07
Esophageal tumor	17	5.39
GEJ Cancer	22	6.98
Level of obstruction		
Non traversable by scope	207	65.71
Traversable by scope	108	34.28

### Type of endoscopic interventions for esophageal cancer patients seen at Lubaga Hospital

Among the 315 patients seen, 134 (42.54%) patients underwent esophageal stenting. 2 (0.77%) had a percutaneous endoscopic gastrostomy (PEG) tube inserted, and 164 patients (52.1%) had no intervention done. Tandem stenting was done in 5.4% of the study group. One patient who had esophageal stenting came back 1 week later having changed their mind about the procedure and requested for the stent to be removed against medical advice. Failure of stenting was in 7 cases in total among which 5 patients had tumor involving the proximal third at the crico-esophageal sphincter, 2 patients had complete esophageal lumen occlusion at the mid-third and distal third of the esophagus (Table 2).

**Table 2:** Interventions for esophageal cancer patients in Lubaga hospital, Kampala

Variable	Frequency (N)	Proportion (%)
Interventions		
Stenting	134	42.54
Stent removal	1	0.32
Tandem stenting	14	5.36
PEG	2	0.77
No stenting	164	52.06

Esophageal region	No. of failed stent procedures	Endoscopic finding
Proximal 1/3	5	Tumor involving the crico-esophageal sphincter
Middle 1/3	1	Complete esophageal lumen occlusion
Distal 1/3	1	Complete esophageal lumen occlusion
Total	7	

Histologically confirmed results were obtained for 227 patients of which 106 patients had adenocarcinoma, 2 patients had adeno-squamous carcinoma and 119 patients had squamous cell carcinoma (Table 3).

**Table 3:** showing esophageal cancer location in relation to the histological type

Region of the esophagus	Histological type of esophageal cancer				
	Adenocarcinoma, n	Adeno-squamous carcinoma, n	Squamous cell carcinoma, n	Missing record, n	Total, n (%)
Proximal 1/3	00	01	37	06	44 (14.0)
Middle 1/3	11	00	69	47	127 (40.3)
Distal 1/3	93	00	00	29	122 (38.7)
Not documented	02	01	13	06	22 (7.0)
Total n (%)	106 (33.7)	02 (0.63)	119 (37.8)	88 (28.0)	315 (100)

## Discussion

Cancer of the esophagus is one of the most aggressive malignancies of the upper digestive tract. It is the ninth most common cancer constituting 3.1% of all cancer cases and the sixth most common cause of mortality constituting 5.5% cancer-related deaths worldwide in 2020. In Uganda, Esophageal cancer is the third commonest malignancy in men and the fourth commonest in women and ranks seventh and accounts for 7.8% of all cancer deaths [10, 11].

Overall, the incidence of esophageal carcinoma increases with age, individuals in their 6th and 7th decades are more affected with an appreciable male predominance. Our findings are similar to results from other studies which showed that majority of the records belong to male patients (67%). This supports the argument that cancer of the esophagus has continued to be a male-dominant disease. A recent study indicates that esophageal cancers are two or three times more common in males than females [12, 13].

The endoscopic findings revealed several tumor descriptions with majorly fungating, friable and polypoid (85.1%). The upper, middle and lower esophagus can be involved. Our study revealed that the majority of the tumors were localized in the middle esophagus (40.3%) followed by the distal esophagus (38.7%) This was slightly different result in comparison with a recent study that reported esophageal cancer commonly arises in the upper and middle esophagus [14].Though both studies agree on the middle esophagus being the predominant site for esophageal cancer.

**Fig. 1:**
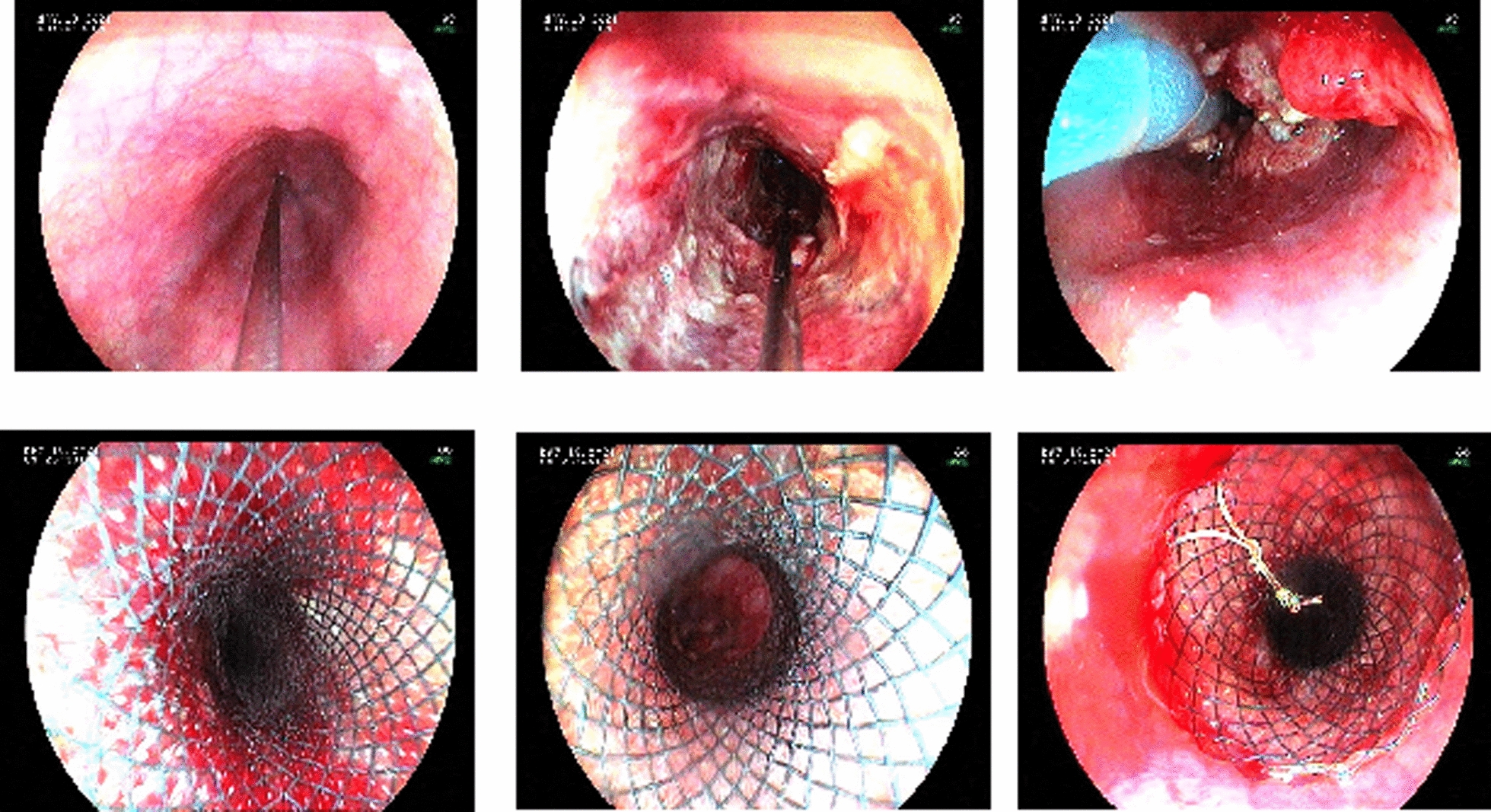
Endoscopic esophageal stenting for unresectable esophageal cancer

Management of esophageal cancer in Uganda remains challenging due to the advanced stage of disease at presentation and poor nutrition status characterized by severe muscle wasting. One of the palliative care modalities is insertion of an esophageal stent to enable feeding (Fig. 1).

In this study 134 of 315 patients underwent primary stenting (42.54%) and 14 underwent tandem (repeat) stenting. The failure rate of stenting was 4.7% due to tumor involving the proximal third at the crico-esophageal sphincter and complete esophageal lumen occlusion at the mid-third and distal third. Despite 207 (66%) of patients presenting with endoscopically non-traversible tumors, we were still able to achieve a stenting success rate of 95.3% even without need for fluoroscopic guidance.

Percutaneous endoscopic gastrostomy (PEG) tubes are a common intervention with the aim of nutritional optimization. PEG allows nutrition, fluids and/or medications to be put directly into the stomach, bypassing the mouth and esophagus. Esophageal stenting is better than PEG insertion in terms of quality of life for patients with unresectable esophageal cancer. However, PEG insertion is cheaper than esophageal stenting. In this study, the two patients who had PEGs inserted were due to the issue of cost. They later changed their minds while still on the ward and had esophageal stenting. These two patients, therefore, underwent initial PEG insertion followed by stenting. Tandem stenting is a repeat stent placement done for previously stented patients who return with dysphagia due to commonly tumour recurrence or tissue overgrowth narrowing the proximal edge of the stent. Tandem stenting was done in 5.4% of the patients.

### Study limitations

Being a retrospective study, we were getting the information from one-page endoscopy reports of these patients. All patients who underwent esophageal stenting had already been histologically confirmed and radiologically staged by the physicians from the referring institutions. The majority of the patients were referred particularly for this specialized service. Therefore, obtaining detailed histology results, staging imaging investigations and detailed endoscopy information was not possible for some patients during the records review in Lubaga Hospital.

## Conclusion

Despite 207 (66%) of patients with advanced esophageal cancer presenting with endoscopically non-traversable tumors, endoscopic stenting was still possible with a technical success rate of 95.3%.

## Data Availability

The datasets used and/or analyzed during the current study are available from the corresponding author, Okello Michael on a reasonable request. All data generated or analyzed during this study are included in this published article.
